# Hyperæmia and Inflammatory Exudations of the Mucous Membrane of the Mouth

**Published:** 1870-04

**Authors:** 


### AMERICAN JOURNAL

OF

### DENTAL SCIENCE.

**Vol. III. THIRD SERIES--APRIL 1870. No. 12.**

### ARTICLE I.

### *Hyperaemia and Inflammatory Exudations of the Mucous Membrane of the Mouth.**

By A German, D. D. S.

I intend to divide this disease into five classes.

1st. Inflammation of the mucous membrane or stomatitis simplex.

*a.* Caused by Vegetable Parasites.

*b.* Caused by Syphilitic Affections.

*c.* Caused by Mercury.

*d.* Caused by Corrosive Remedies.

2d. Stomatitis Vesiculosa et Pustulosa.

3d. Stomatitis Ulcerosa.

4th. Stomatitis Folliculosa et. Aphtosa.

5th. Stomatitis Gangrænosa, Gangraena Infantilis, Noma.

---

* AUTHORITIES:—Buzer Zahnheilekunde: Prof. Albrecht, clinic der Mundkran ksseiten; Bouchut Manuel Pratique des Maladies de nouveau—nes; Vogel—allgemeine Zeintung fur Chirurgie; Reubold, der Soor, Virchow's Archiv xxx, 1864; Smoler Medical Gazette of Vienna 1851; Gazette Med. d Orient; Bauden's Crimeian War; Journal von Graefe und Walter, Pourdes der Noma, ou du Sphacele de la Couche chez les enfant Strasburg, 1848.

### 1st. *Stomatitis Simplex, Pathological Anatomy and Symptoms.*

The mucous membrane of the mouth appears inflamed in separate spots, for instance: at the follicular openings, or the whole surface is in the condition of inflammation, partly deprived of its epithelium. At times increased secretions are observed with abrasions of the surface.

The inflammation of the mucous membrane of the cheek renders mastication difficult, and in children interferes with their suckling, and in consequence of the swelling the shape of the teeth are impressed upon the substance of the cheek. The papillae of the tongue are very frequently injected, and at the margin are indented with the form of the teeth; the taste is generally altered and characterized by intense bitterness.

Should the affection appear on the gums, the sensation of tickling and burning is complained of.

Swelling of mucous membrane of the uvula brings on continued gulping and obstinate coughing.

When the disease becomes extensive, the taste is repugnant, and the breath offensive. Only among very sensitive individuals, and especially among children at the breast, we find general symptoms of headache, fever, restlessness and the other signs of hyperæmia of the brain.

Aetiology. Uncleanliness, parasites, hard sucking, diseased mammaries, cutting of the teeth, are the causes of the affection in suckling children. In other children and adults we also have local irritation like that from foreign bodies, sharp edges of teeth, loose roots, and ill formed clasps, biting of surround ing tissues, salivary calculus, parasites and cauterization, the excessive use of alcohol and tobacco. Constitutional symptoms, such as poison by mercury, iodine, cantharides and phosphorus, also infections in measles, scarlet fever, small pox, syphilis, typhus, etc.

Diagnosis. It is not always possible to determine the origin of the disease from the degree of inflammation; notwithstanding this, the cause can be immediately established by the characteristic clinical symptoms of many forms of stomatitis.

*a. Stomatitis Simplex*

caused by vegetable parasites upon the mucous membrane of the mouth, thrush, scer, muguet. A small confervæ, oidium albicans, generated from an accumulation of round sporules elevated on the surface of the mucous membrane, and between the lamella of the epithelium. It forms sometimes single, sometimes confluent opaque spots, which are easily detached but quickly reproduced. They are most frequently observed on the anterior portion of the fauces, the inner sides of the lips and cheeks, on the gums, and on the margins of the tongue. Occasionally the whole cavity of the mouth and the pharynx are covered with this vegetable growth. The eruption is often found in infants less than a month old, being in them a mild affection; sometimes, however, it appears in adults, generally after some acute or chronic affection, and then characterizes the fatal disease. In both cases this vegetable growth is accompanied by a nauseous stomach. The pains or symptoms, are in general very slight so that infants are seldom prevented from suckling or swallowing. The thrush of adults seldom produces any unpleasantness but is remarkable for the disagreeable odor of cradle milk.

*b. Stomatitis Simplex, caused by Syphilitic Infection.*

It makes its appearance in small oblong light gray spots, which consist of the dried upper layer of the epithelium. Small furrows appear in the epithelial layer, between which the red tissues are again seen. The general location of this affection is principally the mucous membrane of the lips, but sometimes it appears on that of the cheek, the margin of the tongue and the fauces. Swelling of the glands is not a constant symptom.

*c. Stomatitis Simplex, caused by Mercurial Poisoning.*

Before the appearance of salivation, an opaque coloring, shows itself in the mucous membrane of the gums, and contrasts strongly with the red tissues, which is caused by the thickness and opaqueness of the affected epithelium. On the mucous membrane of the cheek, we frequently, at the same time, observe a thin white fur, which is also often called mercurial sore.

*d. Somatitis Simplex, caused by Corrosive Remedies.*

The application of caustic substances to the mucous membrane of the mouth, especially the use of creosote by unskillful persons, are frequent causes of stomatitis. They produce an opaqueness of the upper layer of the epithelium, which is easily detached with the finger, after which a dark colored and bleeding surface is seen; other acids and alcohol produce the same affects as creosote.

Treatment. In treating stomatitis the first step is to remove the irritating causes, should the confervæ be the cause, cleanliness and good nourishment is the best means of treatment. Should the vegetable not be great, the best way is to wash them off with cold water, or paint with a weak sotion of sulph. of copper, or still better apply cerax 1 part and honey 3 parts.

In mercurial stomatitis, the administration of mercury being stopped, the disease will disappear of itself; if not it should then be treated with a weak solution of chlorate of potash. The syphilitic stomatitis being treated by cleanliness, will sometimes spontaneously disappear. Should it not do so, however, an anti-syphilitic treatment will invariably overcome the disease.

2d. *Stomatitis Vesiculosa et Pustulosa.*

The elevation of the epithelium by an accumulation of serum characterizes the vesicular inflammation. Should the serum assume the character of pus, we have pustules and with them a pustular inflammation.

Vesicles are produced by local irritation, cautery &c., especially on the point of the tongue; occasionally no local irritation can be determined, and we then have an eruption, forming on the lips, like herpes. Herpes labialis, olophlyitis prolabiales, as in pneumonia, bronchitis and gastric catarrh, but never, as Prof. Skoda affirms, in typhoid diseases. On the second or third day the opaque, alkaline fluid of the vesicle clears and is gradually absorbed. The residue with the thin skin of the vesicle forms a small yellowish crust, which falls off about the seventh or eighth day, and leaves a minute red spot, which disappears in a short time.

The treatment is expectant and only calls for the administration of quinine, when the erruption is accompanied by neuralgia (as in the interesting case mentioned by Leoni).

Pustules frequently occur in small pox after the use of tartar emetic in large doses, sometimes spontaneously changing from vesicles to the formation of ulcers. Such pustules are of no gravity, and generally disappear without any further treatment.

### 3. *Stomatitis Ulcerosa.*

We distinguish ulcers, produced by local irritation, those by constitutional causes, and others partly by local and partly by constitutional causes.

The most frequent ulcerations produced by local irritations are those caused by sharp edges of teeth, and old roots remaining in the mouth, clasps, and also by perforations of the alveolar processes by the roots of teeth. They are also often caused by mechanical injuries, viz: extractions with the key, from undue pressure, injury of the gum by an antagonistic tooth; cutting of wisdom teeth, and finally isolated teeth in the old.

Chemical and heating applications, are sometimes also the cause of these ulcerations, generally applied through carelessness or with criminal intention. The careless application of creosote to an aching tooth, or the burning of the mucous membrane by the introduction of hot food are frequent causes. The injuries range from the simple abrasion of the surface, to the injury of the deeper tissues, attended by inflammation and swelling of the neighboring glands.

The treatment consists first in the removal of the irritating cause, such as the extraction of the teeth and roots, &c., and the application of mild, lukewarm gargles. Should the di sease not yield, the application of astringent washes is advisable, among others, alum, rhatany, tannin, &c. Fistulous ulcerations are to be opened. In the healing of ulcers of the gums and cheek, which come in contact, the introduction of small pieces of sponge or lint to prevent the adhesion of the touching surfaces, is advisable.

Of the ulcers produced by constitutional causes, the scorbutic, the mercurial, and the syphilitic are the principal.

The ulcers in scorbutic affections are mostly found on the gums, and in very few cases do they attack any other part of the mouth, they are characterized by flat, livid, oedematous edges, a dirty base from which spongy, easily bleeding, granulations spring up. A secretion of disagreeable odor followed by altered blood is also observed to exude. As the disease advances, the teeth become loose, the ulcers assume a grayish hue, and attack the other parts of the mouth; occasionally they ever perforate the cheek and the tonsils; the parotid glands are affected, and it at length ends in caries of the exposed bone.

The first step to be taken in the treatment is the removal of the cause, after which citric acid, green salad, &c., might be administered. During the Crimean war salad made of the (taraxacum officinale linn) was used at meals as a vegetable, being thought an admirable remedy against scorbutis. The administation of good beer, is also recommended. Oppolyer recommends:

℞ Decocti Malti Unc. ½, ad Unc. 6

Fermenti Cereviciae,

Oym. simpl. ana Unc. ½

M. S. A tablespoonful every 2 hours.

In slight cases, locally an astringent wash may form the whole mode of treatment. In graver ones the application of weak solutions of nitric acid, or lapis infernalis suffices. Hippocrates advised the chewing of twigs of myrrh. The ulcers produced by the administration of mercury, first appear on the gums, and the inner walls of the lips, they then spread to the tongue and the cheeks. On the gums they attack the free margins and are covered with a filthy secretion, and are generally quite painful without being particularly different from other ulcerations. But, nevertheless, some symptoms should not be passed over without mention, such as the more or less violent salivation, the highly disagreeable characteristic odor, the effect on the teeth, and other symptoms of mercurial salivation, more so than the simpler ulcerations, is followed by a growing together of the substance of the gums with the cheeks; sometimes perfect atresia oris is established.

The object of the treatment is to get rid of the mercurial salivation, by penciling and cauterizing with nitric acid. The internal treatment consists in the administration of chlorate of potash, one drachm, in divided doses, per day. Adhesion of the substance of the gum and cheek require operations, and are to be kept apart by lint, cauterizing with nitric acid etc., in order to prevent readhesion. It is often very difficult to secure this result as the tendency to reunite is very great, sometimes over-growing the lead wires applied according to Padtorfer.

The syphilitic ulcers are generally situated on the gums; they are, however, sometimes found in the corners of the mouth and on the cheeks.

They form sharp edged ulcers with a fatty looking base to which a yellow secretion adheres. The ulcers are sometimes surrounded by an induration of a dark, frequently coppercolored, inflammation. Sometimes this symptom is, however, not present and the disease cannot be so readily diagnosed without the presence of other syphilitic affections (as condylome), or by the results of a specified treatment. If the disease is not too severe, a simple local treatment will suffice. The use of weak astringent washes and gargles, and the application of lunar caustic are followed by a good result. In most cases, however, in order to thoroughly relieve the disease, an antisyphilitic treatment must be instituted. In such cases, with adults, the administration of the hydrargyrum Iodatum gr. ss. pr. die. is recommended. In children a dose of calomel gr. $\frac{1}{4}$. night and morning, or the application to the skin of ung. ciner. gr. 5-8. at night, followed by a warm bath in the morning is thought to be an efficient mode of treatment.

Ulcers produced partly by local and partly by constitutional influence are presented by the

### 4. *Stomatitis folliculosa. s. aphthosa.*

The follicular inflammations of the mucous membrane of the mouth are most frequently found in children, but sometimes also in adults. They generally attack the mucus follicles of the mouth. As a rule the sore begins by the appearance of an elevated pale looking vesicle, of a white or yellowish color, which, after bursting, exposes a small ulcer (Aphtha) with red edges and a yellowish gray base. In about a week or ten days this ulcer cleans up and cicatrizes. Their favorite places of attack are the inner surface of the lower lip and the cheeks, the margins of the tongue and the mucous membrane of the gums.

The causes of this disease are very obscure. As one-half of the number of all observations fall in the second year of life, and as fully two thirds of them are between the ages of 5 and 30 months, it would point to the time of the first dentition. But we also have them in typhus and puerperal fevers, and in the course of many severe acute diseases. This disease itself involves no unfavorable prognosis. The symptoms vary according to the number of the ulcers. In little children the appearance of a great number of these sores produces fever, gastro-intestinal catarrh, swelling of the neighboring lymphatic gland, etc., in adults they are very frequently the cause of excesive burning pain, and when the disease lasts a longer time, disturbance of nutrition. The best means of cure is the application of lunar caustic.

5. *Stomatitis gangraenosa, Gangraena Infantilis, Noma.*

The gangrenous mouth, very inapropriately called noma, since the time of the Dutch physician Van der Voorde, in the 17th century, consists in a very rapidly increasing gangrene of a part of the soft tissues of the mouth, the formation of a filthy exudation, indicative of the fast destruction of the tissues, and the consequent general symptoms, frequently ending in death.

The disease commences with the formation of a livid red spot on some part of the mucous membrane of the cheek, most frequently on the left side, at which spot the epithelium rises into a small vesicle. At the same time the cheek begins to swell, and a central cone is formed surrounded by an elastic, and less pliable tissue. The cheek at this place is tense and shining, as if covered with oil, but nevertheless not so hard and unyielding as to prevent the mouth being opened for an examination.

In the middle of the swelling, a spot of a dark red color now begins to grow darker, and to increase in circumference, and the swelling gives way to a shaggy, pulpy, dark brown mass. The corresponding spot in the mouth is changed into a deep ulcer, with irregular jagged edges and a dirty dark brown base, the neighboring gum is in a similar condition, the teeth are loosened and fall out one after another. The salivary secretion is increased, while the exudation, at first clear, becomes cloudy and of an offensive odor, and finally the sympathetic glands swell more and more.

As a rule the pains are not very severe, the patients feel sleepy, and death is very easy, often occuring before the gangrenous part sloughs off.

This terrible disease is generally only found in children between the ages of two and five years, occuring more frequently in girls than in boys. More cases occur in cold climates than in warmer ones. Poor living and severe attacks of illness, principally of measles, typhus, and tuberculous affections appear to have a decided tendency to produce this disease. The real cause of this affection is not known.

Treatment, 1. Cauterize suspicious looking ulcers in time, with lunar caustic or nitric acid.

The following wash has been used with success:

℞. Bromi Puri, gr. vj.
Kali hydrobromi, gr. xxiv.
Aquae destillatae, ℥ ss.
M. S. To be sprinkled on ulcerations.

2. Endeavor to keep the mouth and ulcer clean, by the application of lukewarm aromatic washes and gargles as for instance:

℞ Infusi Specier, aromatic de ʒ ij.
Creosote puri ggt. j.
M. S. Gargle.

3. Administer internally Potass. chlor. and good nourishing food.

---

